# Socioeconomic inequity in inpatient service utilization based on need among internal migrants: evidence from 2014 national cross-sectional survey in China

**DOI:** 10.1186/s12913-020-05843-w

**Published:** 2020-10-27

**Authors:** Yi Wang, Zhengyue Jing, Lulu Ding, Xue Tang, Yuejing Feng, Jie Li, Zhuo Chen, Chengchao Zhou

**Affiliations:** 1 Centre for Health Management and Policy Research, School of Public Health, Cheeloo College of Medicine, Shandong University, Jinan, 250012 China; 2 NHC Key Lab of Health Economics and Policy Research (Shandong University), Jinan, 250012 China; 3 College of Public Health, University of Georgia, Athens, GA 30606 USA; 4 School of Economics, University of Nottingham, Ningbo, China

**Keywords:** Migrants, Socioeconomic status (SES), Inpatient service utilization, Concentration index, Blinder-Oaxaca decomposition, China

## Abstract

**Background:**

Providing equal treatment for those who have the same need for healthcare, regardless of their socioeconomic and cultural background, has become a shared goal among policymakers who strive to improve healthcare. This study aims to identify the socioeconomic status (SES) inequities in inpatient service utilization based on need among migrants by using a nationally representative study in China.

**Methods:**

The data used in this study was derived from the 2014 National Internal Migrant Population Dynamic Monitoring Survey collected by the National Health Commission of China. The sampling frame for this study was taken using the stratified multistage random sampling method. All provincial urban belt and key cities were stratified, and 119 strata were finally determined. We used logistic regression method and Blinder-Oaxaca decomposition and calculated the concentration index to measure inequities of SES in inpatient service utilization based on need. Sample weights provided in the survey were applied in all the analysis and all standard errors in this study were clustered at the strata level.

**Results:**

Of the total internal migrants, 18.75% unmet the inpatient service need. Results showed that inpatient service utilization concentrated among high-SES migrants (Concentration Index: 0.036, *p* < 0.001) and the decomposition results suggested that about 44.16% of the total SES gap in inpatient service utilization could be attributed to the gradient effect. After adjusting for other confounding variables, those had high school degree and university degree were more likely to meet the inpatient services need, and the OR values were 1.48 (95% CI 1.07, 2.03, *p* = 0.017) and 2.04 (95% CI 1.45, 2.88, *p* = 0.001), respectively. The OR values for Quartile 3 and Quartile 4 income groups was 1.28 (95% CI 1.01, 1.62, *p* = 0.044) and 1.37 (95% CI 1.02, 1.83, *p* = 0.035), respectively.

**Conclusion:**

This study observed an inequity in inpatient service utilization where the utilization concentrates among high SES migrants. It is important for policy makers to be aware of them and more intervention should be conducted.

**Supplementary Information:**

**Supplementary information** accompanies this paper at 10.1186/s12913-020-05843-w.

## Background

According to the World Health Organization (WHO) [1], the key goal of the universal health coverage (UHC) is to ensure that everyone receive the health care they need. Providing equal treatment for those who have the same need for healthcare, regardless of their socioeconomic and cultural background, has become a shared goal among policymakers who strive to improve healthcare. However, millions of people, especially migrants, do not have the adequate access to health-care services they need [1]. Migrants face many obstacles in accessing essential health care services due to factors such as language barriers, a lack of inclusive health policies, and inaccessible public services [2]. The WHO has been promoting the health of migrants and committed to adequately address health needs for migrants. A WHO framework for migrant health has recognized the urgent need for the health sector to address the impact of migration on health effectively [2].

China has experienced the largest migration during the past three decades, with the number of migrants increased from 230 million in 2011 to 244 million in 2017, which constituted 18% of the total population of China [3]. Internal migrants, in Chinese literally “floating population”, which is defined as those who have left their hometowns to live and work in a new place for more than 1 month but do not have a local ‘Hukou’ (registered residence) at the new location [4]. Since 1980s, the rate of urbanization has increased dramatically in China which is due to internal rural-to-urban migration [5]. However, migrants are known to be marginalized in China, because of the Hukou system. Although the internal migrants have made an important contribution to urban economic growth and social stability, their health status and health service utilization have not received due attention. Comparing with local residents, the migrants face higher unmet health care need and poorer quality care [2, 6]. The challenge for China is how to promote economic growth and develop wealth while reducing inequality among migrants. Addressing the health care needs of migrants can improve their health status, facilitate social integration, and contribute to economic development [7]. During the past two decades, China implemented several national healthcare development plans [8] to improve healthcare access and equality with many challenges and successes. National Health Commission of the People’s Republic of China (NHC-PRC) has started an initiative called ‘Equal Access to Public Health Services among Migrants’ since 2013, to improve access to public health service especially the utilization of health service.

Equity in health can only be attained if persons with the same level of healthcare needs receive equal level of care, regardless of their socioeconomic status (SES). However, little evidence exists on the SES inequities in health service utilization among internal migrants in China. To date, published studies have mostly been divided into three categories. The first is about the difference and comparison of the utilization of health services between the migrants and the local residents [6, 9, 10]. Second, most studies on the internal migrants are based on regional data [11–15], and there were few studies using a nation-wide data about the migrants. More importantly, most of the studies focused on the influencing factors of the utilization of health services of the migrants, but few explored from the perspective of health need [13, 16, 17]. Although SES equity is very important for migrants in access to healthcare services, there is a shortage of studies explored the SES inequities in inpatient service utilization among internal migrants in China, especially based on the need of inpatient service. In order to fill these gaps, this study was performed to explore the SES inequities in inpatient service utilization based on need among the internal migrants in China, in order to quantify SES roles in healthcare utilization inequity as a guide for health policy makers and draw public policy implications to further reform the health care systems.

## Methods

### Study design and data

**Fig. 1:**
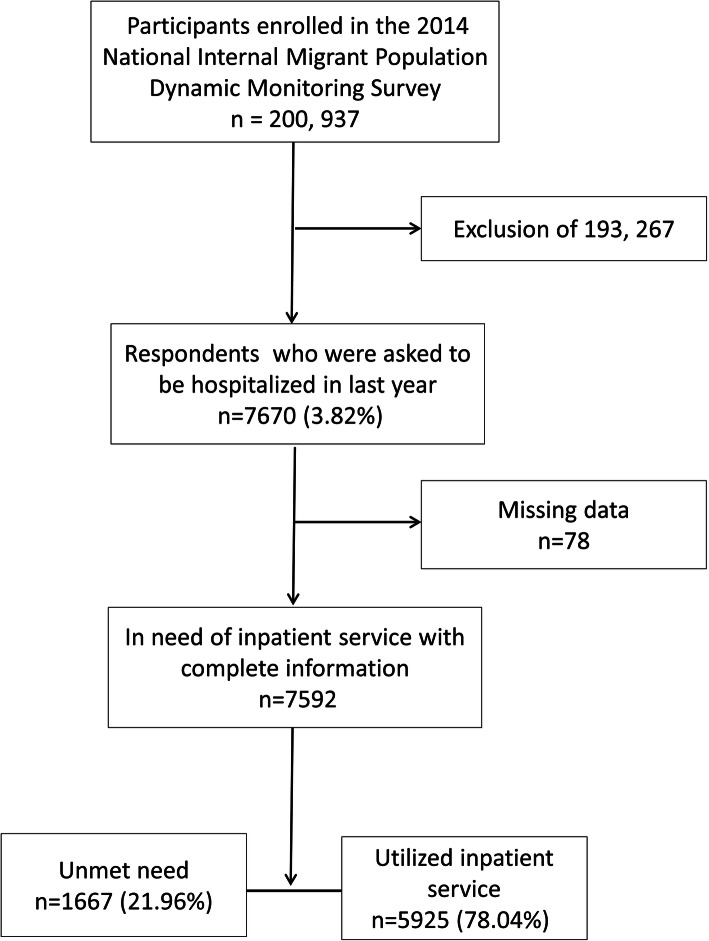
Flow chart of sample selection

The data used in this study was derived from the 2014 National Internal Migrant Population Dynamic Monitoring Survey [18], which covered 348 cities in 32 provincial units and collected by the National Health Commission of China. The purpose of the survey was to investigate the utilization of health services among internal migrants. The sampling frame for this study was taken using the stratified multistage random sampling method by probability proportional to size (PPS) approach. All respondents in this study were aged 15–59 years who had been living in local residence without the ‘Hukou’ for more than 1 month, including migrants from both rural and urban areas. For more details on sampling, design and approvals of the survey, please refer to an earlier study [19]. The detailed sampling process was shown in Fig. 1**.** Finally, a total of 7592 migrants with inpatient service need were included in this nationally representative analysis.

### Dependent variable

In this study, the estimation of the need of inpatient services is based on doctor’s recommendations. Thus, migrants’ inpatient service need was measured by questions about whether they were asked to be hospitalized by a doctor’s diagnosis. Based on the inpatient service need, the outcome was categorized into unmet inpatient services need and met need. The unmet need for inpatient service referred to the proportion migrants who were asked to be hospitalized by a doctor but did not utilize it. The key independent variable was socioeconomic status (SES). American sociologist Duncan pointed out that income and education can directly represent socioeconomic status, that is, income represents economic status, and education represents social status [20]. Li et al. [21] also suggested that the SES estimated based on education and income was largely consistent with people’s subjective evaluation of occupation prestige. Consequently, we used educational attainment (primary school or below, middle school, high school, and university or above) and economic level (measured by household income per month. Quartile 1 was the poorest and Quartile 4 was the richest) to present SES. In this study, we assessed the SES in two ways. First, in order to compare the inequity of the high-low SES in inpatient service utilization from a macro perspective, we integrated the educational level and economic status (household income per month) into a single SES index using principal component analysis (PCA) [22] method (details see in Appendix Table A1). Then, we used the two specific indicators to show the associations between SES and inpatient service utilization.

### Independent variables

The confounders adjusted in this study are based on previous literature on health service utilization and Andersen health service utilization model [23–25]. The selection principle of control variables is related to both core explanatory variables (SES) and dependent variables (inpatient services). According to Andersen model, individuals deciding whether or not to use health services are mainly influenced by three factors: predisposing characteristics, enabling resources and actual need for care. Based on previous studies, predisposing factors mainly include gender, age, marital status, number of children, and duration of migration etc. Enabling resources mainly include the individual’s SES, ethnic group, health insurance status, health records, and hukou etc. Thus, control variables in this study include gender, age, marital status (married or single), number of children, ethnic group (Han or ethnic minority), establishment of health records, Hukou types (urban or rural), health insurance, movement area (across province, city or county), duration of migration, region (east, central or west), and willingness for long-term residence of more than 5 years (yes, no, and not decided yet). Types of health insurance were divided into four subgroups: no health insurance, having New Rural Cooperative Medical Scheme (NCMS), having Urban Employee Basic Medical Insurance (UEBMI), and having Urban Resident Basic Medical Insurance (URBMI). Movement area was categorized into three types: migration across provinces; migration across prefectural cities but within a province and migration across counties but within a prefectural city. All the control variables were available through the 2014 National Internal Migrant Population Dynamic Monitoring Survey. In order to avoid collinearity caused by the possible highly correlation between control variables and SES, regression diagnosis was carried out. The correlation matrix among SES and confounding variables have shown that the largest absolute value of the correlation coefficient was only 0.4, showing a weak correlation. Further, we calculated the variance inflation factors and found all the factors were no more than 2, a trivial amount, suggesting that the correlation between SES and covariates is not a serious problem.

### Analytical methods

Data analyses were conducted by using the STATA 14.2 (Stata Corporation, College Station, TX, USA). Descriptive analyses were performed to compare the inpatient service utilization across different subgroups of the participants using t-test or chi-square test as appropriate and reported their *p*-values. Sample weights were applied in all the analysis to represent the China population. To avoid possible regional variation in this study, we controlled regional variable (east, central or west) in all regressions, and all standard errors were clustered at the strata level. To be specific, based on the study design of this survey, the weights of this study are only derived from strata and non-response weight, and all provincial urban belt and key cities were stratified, and 119 strata were finally determined. For example, Beijing was divided into seven strata, namely Chaoyang district, Haidian District, Fengtai District, Daxing District and etc. We used survey commands (*svy*) for estimates of sample weighting and clustering. The *pweight* command was used to define the sample weights and *strata* command was used to define strata in order to generate clustering robust standard errors. These commands fits statistical models for complex survey data by adjusting the results of a command for survey settings identified by svyset. Thus, all standard errors in this study were clustered at the strata level in order to eliminate the variations within strata level.

First, we estimated the concentration index (CI) and constructed a concentration curve (CC) to illustrate inequity in unmet inpatient service need among migrants. The CC graphs the cumulative percentage of the sample on the x-axis, ranked by SES index, beginning with the lowest. CI was used to quantify the magnitude of inequity in unmet need and corresponds to twice the area between the CC and the 45° line [26]. CC runs from − 1 (over-diagonal) to + 1 (under-diagonal), indicating whether the unmet inpatient service need is concentrated among the low-SES (CI < 0), the high-SES (CI > 0), or equally distributed (CI = 0) [27].

The concentration index *C*_*M*_ was calculated by the following formula:

1 $$ {C}_M=\frac{2}{N\overline{y}}\sum \limits_{i=1}^N\left({y}_i-\overline{y}\right)\left({R}_i-\frac{1}{2}\right) $$

2 $$ =\frac{2}{\overline{y}}{\operatorname{cov}}_w\left({y}_i,{R}_i\right) $$

Where $\overline{\ y}$ stands for the mean of *y*, *y*_*i*_ is the measure of unmet inpatient service need of *i*th individual, *R*_*i*_ denotes the fractional rank of the *i*th individual in the SES index, and cov_*w*_ is the covariance with sampling probability weights. The concentration index and the associated *p*-values were obtained by the delta method [28]. If the *C*_*M*_ is significantly smaller than 0, low SES individuals are more likely to have unmet inpatient service need, and vice versa [29].

Then, we adopted logistic regression method to investigate the SES disparities in multivariate analyses adjusted for confounding variables. Those who received inpatient services were defined as the reference group. In model 1, we examined the association between SES and inpatient service utilization without control variables. In model 2, we controlled for potential confounding factors and estimated the adjusted odds ratio and the 95% confidence intervals. The model was specified as:

3 $$ \mathrm{Logit}\left(\frac{p_i}{1-{p}_i}\right)={\beta}_0+{\beta}_1\ast {\mathrm{SES}}_{\mathrm{i}}+{\beta}_2\ast {\mathrm{C}}_{\mathrm{i}}+\upepsilon $$

Where *p*_*i*_ represented the probability of inpatient service utilization; SES_i_ represented the socioeconomic status of *i*th individual; C_i_ indicated the confounding variables; Coefficients *β*_0_ and *β*_1_ represented intercept and SES inequalities, respectively; ϵ indicated error terms; OR indicated Odds Ratio.

Finally, the decomposition of the gap in inpatient service use between the high and low SES migrants was assessed using the Blinder-Oaxaca (BO) decomposition method. The BO decomposition method was originally developed to explain wage gaps between whites and blacks and between men and women since the seminal work of Oaxaca and Blinder in the early 1970s [30, 31]. The BO decomposition [32] was a counterfactual method with an assumption that “what the probability of unmet inpatient service need would be if low SES migrants had the same characteristics as their high SES counterparts”. In this part, SES was created using a median split with low SES categorized as below the median of SES index total score and high SES categorized as above the median. Based on it, the SES inequity was divided into two parts by using BO decomposition as followed:

4 $$ \mathrm{E}\left({P}_h-{P}_l\right)=\left(\mathrm{E}\left[{Z}_h\right]-E\left[{Z}_l\right]\right){\beta}_l+E\left[{Z}_h\right]\left({\beta}_h-{\beta}_l\right) $$

Where *l* represented low SES migrants and *h* represented high SES migrants; Z represented all the independent variables in our study; *β* represented the estimated coefficients. The first term in Eq. (4) corresponded to the proportion of the gap in outcomes between two groups that were accounted for by group differences in the distribution of observable characteristics, it indicated “endowments effect”, this section caused by differences in migrants’ characteristics, so it was also called explained component; while the second term was “gradient effect” which traced the differences attributable to the effect of the variables, this part aroused because of the differences in SES effects or attributed to “discrimination”. Decomposing SES differences in inpatient service utilization into endowments and gradient effects has strong policy implications since the evidence of gradient effect would reflect that high-low SES migrants endowed with the same characteristics do not enjoy the same level of inpatient service.

## Results

The participants enrolled in 2014 National Internal Migrant Population Dynamic Monitoring Survey were 200,937, of which, around 3.82% had the inpatient service need. According to the Table 1, the total number of the migrants who needed inpatient service diagnosed by doctors was 7592, of which, 1667 (18.75% of total population) did not use the inpatient services (unmet inpatient service need) and 5925 (81.25% of total population) had used the inpatient services. Of the 7592 participants, about two-thirds (*n* = 5461) were female. The mean age was 32 years old. Most of the migrants had middles school degree (45.65% of total population), were Han Chinese (92.92% of total population) and had been married (94.70% of total population). About 97.31% had at least one child; 82.46% were registered as having a rural ‘Hukou’ and 76.90% had established the health records in the local residence. Regarding health insurance, 50.90% were covered by the NCMS, 6.41 and 25.19% were covered by the URBMI and UEBMI, respectively, while 17.50% had no social health insurance. The majority of the migrants was across province migration (66.87%) and has willingness for long-term residence (63.02%). In terms of geographic region, the proportion of migrants in eastern region was the highest (77.90%), followed by western (15.05%), while central regions had the lowest proportion (7.05%). In general, we found that there were statistically significant differences in socioeconomic status, gender, age, marital status, number of children, duration of migration, and regions by whether met inpatient services needs using chi-square tests.

**Table 1:** Characteristics of the migrants who need to be hospitalized by doctor’s diagnosis, China (*n* = 7592)

Characteristics	Total N (%)	Inpatient services		
		Met need N (%)	Unmet need N (%)	***p-***value
**Total**	7592	5925 (81.25)	1667 (18.75)	
***Socioeconomic status***				
**Educational attainment**				< 0.001
Primary school or below	1106 (12.48)	721 (10.66)	385 (20.37)	
Middle school	3544 (45.65)	2725 (45.17)	819 (47.74)	
High school	1499 (21.42)	1226 (22.12)	273 (18.37)	
University or above	1443 (20.45)	1253 (22.05)	190 (13.52)	
**Economic status**				< 0.001
Quartile 1	1927 (18.26)	1363 (16.93)	564 (23.99)	
Quartile 2	2227 (26.27)	1724 (25.70)	503 (28.74)	
Quartile 3	1565 (22.21)	1286 (22.97)	279 (18.94)	
Quartile 4	1873 (33.26)	1552 (34.40)	321 (28.34)	
***Control variables***				
**Gender**				< 0.001
Female	5461 (78.82)	4670 (83.87)	791 (56.92)	
Male	2131 (21.18)	1255 (16.13)	876 (43.08)	
**Age**	32.31	31.25	36.90	< 0.001
**Marital status**				< 0.001
Married	7120 (94.70)	5649 (96.21)	1471 (88.19)	
Single	472 (5.30)	276 (3.79)	196 (11.81)	
**Number of children**				< 0.001
0	195 (2.69)	126 (2.01)	69 (5.60)	
1	4070 (50.72)	3340 (53.04)	730 (40.69)	
≧2	3327 (46.59)	2459 (44.95)	868 (53.71)	
**Ethnic group**				0.943
Han	6915 (92.92)	5396 (92.91)	1519 (92.99)	
Ethnic minority	677 (7.08)	529 (7.09)	148 (7.01)	
**Health records**				0.103
Yes	5648 (76.90)	4396 (76.25)	1252 (79.74)	
No	1944 (23.10)	1529 (23.75)	415 (20.26)	
**Hukou**				0.423
Urban	1301 (17.54)	1041 (17.84)	260 (16.22)	
Rural	6291 (82.46)	4884 (82.16)	1407 (83.78)	
**Health insurance**				0.318
No insurance	1066 (17.50)	844 (18.00)	222 (15.34)	
NCMS	4447 (50.90)	3433 (50.04)	1014 (54.60)	
URBMI	551 (6.41)	410 (6.51)	141 (5.98)	
UEBMI	1528 (25.19)	1238 (25.45)	290 (24.08)	
**Movement area**				0.077
Across province	3650 (66.87)	2867 (67.60)	783 (63.71)	
Across city	2330 (23.23)	1838 (23.00)	492 (24.23)	
Across county	1612 (9.90)	1220 (9.40)	392 (12.05)	
**Duration of migration (year),**	4.30	3.98	5.66	< 0.001
**Plans for long-term residence (> 5 years)**				0.195
Yes	5072 (63.02)	3940 (62.59)	1132 (64.85)	
No	740 (9.60)	565 (9.31)	175 (10.84)	
Not decided yet	1780 (27.39)	1420 (28.10)	360 (24.31)	
**Regions**				< 0.001
East	3286 (77.90)	2716 (79.23)	570 (72.12)	
Central	1632 (7.05)	1219 (6.70)	413 (8.61)	
West	2674 (15.05)	1990 (14.07)	684 (19.27)	

*The percent in parentheses were weighted with sampling weights provided in the survey; NCMS* New Rural Cooperative Medical Scheme, *UEBMI* Urban Employee Basic Medical Insurance, *URBMI* Urban Resident Basic Medical Insurance*. Quartile 1 was the poorest and Quartile 4 was the richest*

**Fig. 2:**
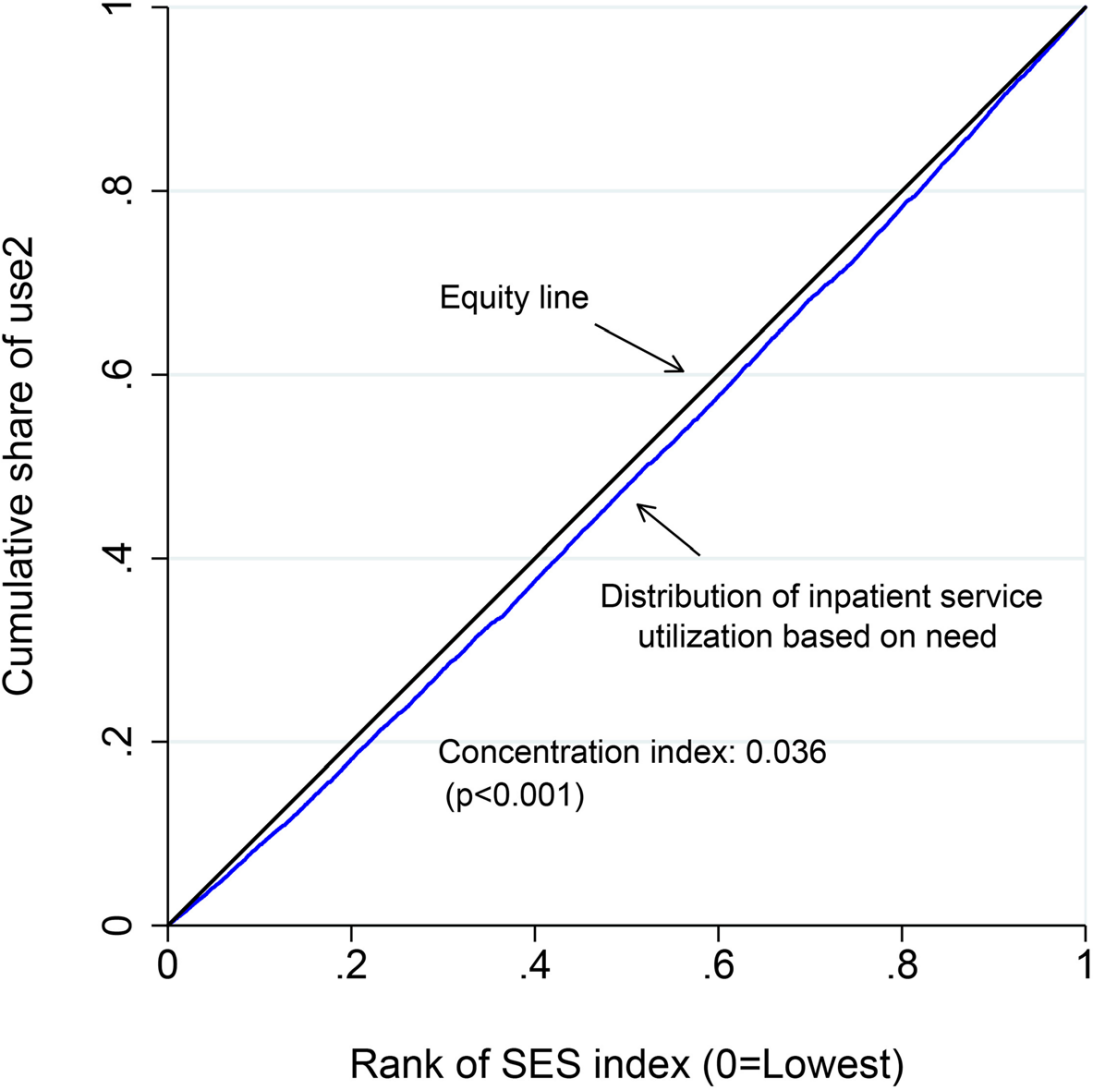
Concentration curves for probability of inpatient service use among migrants

Figure 2 plotted the concentration curves for probability of inpatient service utilization among migrants in the previous 12 month. A significant distribution of inpatient service utilization based on need concentrated among high-SES migrants was observed (CI: 0.036, *P* < 0.001).

Table 2 showed the association between SES indicators and met inpatient healthcare services need among migrants. Model 1 presented the disparities in met inpatient services need in different SES without covariate adjustment. Compared with migrants who had primary school or below degree, those had middle school degree, high school degree, and university degree were more likely to meet the inpatient services need, and the OR values were 1.72 (95% CI 1.33, 2.22, *p* < 0.001), 2.15 (95% CI 1.63, 2.85, *p* < 0.001), and 2.76 (95% CI 2.11, 3.62, *p* < 0.001), respectively. Regarding economic status, compared with the migrants from lowest economic group, the odds of inpatient service utilization when needed were significantly higher among those with higher economic group. The OR values for each income group from the Quartile 2 to the Quartile 4 was 1.41 (95% CI 1.14, 1.76, *p* = 0.002), 1.62 (95% CI 1.34, 1.97, *p* < 0.001), and 1.67 (95% CI 1.25, 2.22, *p* < 0.001), respectively. In Model 2, we included the two SES indicators at the same time and adjusted for other confounding variables. Both the coefficients of education and income were attenuated compared with model 1. Specifically, compared with migrants who had primary school or below degree, those had high school degree and university degree were more likely to meet the inpatient services need, and the OR values were 1.48 (95% CI 1.07, 2.03, *p* = 0.017) and 2.04 (95% CI 1.45, 2.88, *p* = 0.001), respectively. For migrants who had middles school degree, the OR was greater than 1, but it is not statistically significant. Regarding economic status, after adjusting for other confounding variables, the OR values for Quartile 3 groups and Quartile 4 groups was 1.28 (95% CI 1.01, 1.62, *p* = 0.044) and 1.37 (95% CI 1.02, 1.83, *p* = 0.035), respectively.

**Table 2:** Association between socioeconomic status and receivers of inpatient services among migrants who need them, China

Characteristics	Model 1 (No covariates)			Model 2 (Covariates)		
	OR (SE)	95% CI	*P*	OR (SE)	95%CI	*P*
***Socioeconomic status***						
**Educational attainment**						
Primary school or below	Ref.			Ref.		
Middle school	1.72 (0.23)	1.33, 2.22	< 0.001	1.21 (0.16)	0.93, 1.57	0.154
High school	2.15 (0.31)	1.63, 2.85	< 0.001	1.48 (0.24)	1.07, 2.03	0.017
University or above	2.76 (0.38)	2.11, 3.62	< 0.001	2.04 (0.36)	1.45, 2.88	0.001
**Economic status**						
Quartile 1	Ref.			Ref.		
Quartile 2	1.41 (0.16)	1.14, 1.76	0.002	1.15 (0.14)	0.90, 1.47	0.250
Quartile 3	1.62 (0.16)	1.34, 1.97	< 0.001	1.28 (0.16)	1.01, 1.62	0.044
Quartile 4	1.67 (0.24)	1.25, 2.22	< 0.001	1.37 (0.20)	1.02, 1.83	0.035

*Standard error in parentheses all clustered at strata; Sample weights applied; CI indicated confidence interval; Model 2 were adjusted for gender, age, marital status, number of children, ethnic group, health record, Hukou type, health insurance, movement area, duration of migration and willingness for long-term residence of more than 5 years and region*

Table 3 presented the BO decomposition results. The probabilities of inpatient service utilization when needed were 84.8% for high-SES migrants and 77.2% for low-SES. Both endowments effect and gradient effect were significant in logistic decompositions, 55.84% of the gap between the two groups could be attributed to differences in the distribution of explanatory variables included in the model. About 44.16% of the total SES difference in inpatient service utilization could be attributed to gradient effect.

**Table 3:** BO decomposition of the inpatient service utilization when needed among migrants (*n =* 7592)

	Coef. (SE)	95% CI	Contrib. (%)	P
**Predicted probability**				
High SES	0.848 (0.017)	0.816, 0.881	–	< 0.001
Low SES	0.772 (0.014)	0.744, 0.779	–	< 0.001
**Difference in predicted probability**				
Total gap	0.077 (0.016)	0.046, 0.107	100	< 0.001
Due to endowments effect	0.043 (0.011)	0.022, 0.064	55.84	< 0.001
Due to gradient effect	0.034 (0.013)	0.009, 0.059	44.16	0.008

*Regressions and decompositions are weighted with sampling weights provided in the survey. Standard error in parentheses all clustered at strata*

Table 4 showed the composition of the self-reported reasons for unmet inpatient service need, of which the most important was feeling unnecessary (41.0%), followed by the economic difficulties (29.5%).

**Table 4:** Self-reported reasons for unmet inpatient service need among the migrants

Reasons	%
Feeling unnecessary	41.0
Economic difficulty	29.5
Have no time	16.9
No one to take care of	7.1
No effective treatment	2.3
Others	2.1
Lack of hospital beds	1.0

*The percent were weighted with sampling weights provided in the survey*

## Discussion

Healthcare utilization based on need is a key indicator to assess the operation of a country’s healthcare system, and any barriers of access to healthcare should be identified and then eliminated [33]. It is important to assess equity in meeting health services need rather than accessing to healthcare, since access simply denotes an opportunity to receive healthcare, while meeting need mean utilizing the opportunity. One of the objectives of UHC is equity in access to healthcare services, which means ‘everyone who needs these services should get them, not just those who can pay for them [34]. Analyzing the SES inequities in inpatient service utilization based on need among the migrants is vital to develop targeting measures, so as to better meet the health services need of the migrants. Using the National Internal Migrant Population Dynamic Monitoring Survey dataset in 2014, we found that the rate of unmet inpatient service need among migrants was 21.96%, which was higher than 17.1% of general population [35], implying the migrants still face many barriers in accessing essential health care than the local residents. CI has been widely used in the health inequity literature. This study found that CI was significantly larger than 0 and the CC lying over the line of equality, meaning inpatient service utilization concentrated more among the high SES group. Socioeconomic inequality in the use of healthcare, i.e., the high SES group having a higher probability of healthcare utilization when needed, is a persistent in low- and middle- income countries [36]. Our results are similar to previous studies on general healthcare utilization in China [37, 38].

We found the two SES indicators, including economic status and educational attainment, are statistically significant. Our study indicated that low economic status of internal migrants was a key barrier to accessing inpatient service. Compared with those in the low-economic status group, internal migrants with higher economic status were more likely to utilize inpatient service when they had an inpatient service need*,* which was consistent with the second self-reported reason shown in Table 4 (economic difficulty, 29.5%) for unmet inpatient service need among internal migrant. Previous studies have shown that the risk of unmet inpatient service of the poor people was significantly higher than that of non-poor people [24], both in the permanent residents and the migrants [4, 39] . There are several possible reasons for this finding. First, migrants with higher economic status in China have higher payment capacity, and hence, they were more likely to use inpatient services when in need. In contrast, most of the migrants abandon hospitalization because of poor affordability [40]. Second, most of those with low economic status are those rural-to-urban migrants. The primary goal of migration among this population is in search of economic opportunities in urban areas. Thus, they tend to focus on their economic conditions only, and usually do not prioritize their own health [2]. Even if they needed inpatient health services, going to hospital would cost them a fortune. Despite the nearly universal medical insurance coverage in China, economic status remains the dominant barrier to healthcare services utilization [25, 41, 42], including outpatient and inpatient services, and lead to inequity in general health care utilization [38, 40, 43]. This phenomenon is even more severe among the internal migrants. This study also suggests that low educational attainment is associated with unmet inpatient service need among internal migrants, which is consistent with other studies [16, 44–46]. One possible interpretation for this finding is that the internal migrants with higher education usually have more knowledge and higher awareness about the importance of inpatient service use, and this may facilitate their utilization of inpatient services when they have a need.

Consistently, the results of BO decomposition also show that migrants with high SES have higher probability of meeting inpatient service need. About 55.84% gap in unmet inpatient service need between low and high SES can be explained by difference in the levels of observable characteristics. The “gradient effect”, which is considered as “discrimination” in previous studies, reflects inequity here. The decomposition results suggest that about 44.16% of the total SES gap in inpatient service utilization could be attributed to the gradient effect. Namely, SES inequity could account for around 44% in unmet inpatient service need among migrants. Migrants with lower SES may choose to delay or resist the need of inpatient services since meeting the need of inpatient services often means high medical expenses. Improving social and economic resources of low SES migrants would be helpful for reducing the barriers of unmet inpatient need. To be specific, policy makers should develop pro-poor health insurance scheme in migrants with low economic status. Also, future interventions might consider using health education focusing on migrants with low level of education. It is worth mentioning that popular and easy ways should be conducted to intervene for migrants with low educational attainment and improve their use of inpatient service when in need. For example, a better form of health education on migrants is peer education. Those low education migrants with similar age profile, gender and economic status can have common topics of discussion, and thus share information, so as to amplify the effect of “peer effect”.

Although previous studies have shown that high-SES is a protective factor in using public health service among the migrants [12, 16, 17, 47–49], few explored from the perspective of inpatient services need among internal migrants in China. The present study also has several limitations. First, the utilization of inpatient services is self-reported, thus, recall bias might exist. Second, due to the lack of the information on the use of outpatient services, we cannot analyze the utilization of full health services of migrants. Third, most of migrants using inpatient services are female. The reasons of inpatient services for female may be childbirth, which might lead to some bias. Finally, although we have controlled the inpatient service need, we are unable to further adjust for health-related variables due to the limitations of our dataset, such as comorbidities or clinical risk factors.

## Conclusion

In conclusion, this study observes an inequity in meeting inpatient service needs among migrants where the utilization concentrates among those with high SES. The migrants with higher economic status and educational attainment are both more inclined to utilize inpatient services when needed. The findings imply that more interventions should be conducted. A mix of pro-poor health insurance schemes and a post-discharge medical finance aids might be useful to improve the inpatient service use when needed among the migrants with low economic status. In addition, a health education about the importance of inpatient service use when needed for those with low educational attainment might be helpful.

## Supplementary Information

**Additional file 1.**

## Data Availability

The datasets used and/or analysed during the current study available from the corresponding author on reasonable request.

## Abbreviations

SES: Socioeconomic status
UHC: Universal health coverage
WHO: World Health Organization
NHC-PRC: National Health Commission of the People’s Republic of China
PPS: Probability proportional to size
PCA: Principal Component Analysis
NCMS: New Rural Cooperative Medical Scheme
UEBMI: Urban Employee Basic Medical Insurance
URBMI: Urban Resident Basic Medical Insurance
CI: Concentration Index
CC: Concentration Curve
BO: Blinder-Oaxaca
OR: Odds Ratio
