# Supplementary material for: Socioeconomic inequity in inpatient service utilization based on need among internal migrants: evidence from 2014 national cross-sectional survey in China
Source: BMC Health Serv Res. 2020 Oct 27;20:984. doi: 10.1186/s12913-020-05843-w (PMC7590715; doi:10.1186/s12913-020-05843-w)
Supplement: Supplementary file 1 — Additional file 1. [file 12913_2020_5843_MOESM1_ESM.docx]

**Appendix**

**Table A1** Principal component analysis for the SES index score

|  | Principal component 1 |
| --- | --- |
| Eigenvalue | 1.192 |
| Proportion of variance (%) | 59.58 |
| Factor loadings |  |
| Household income per month | 0.7719 |
| Education | 0.7719 |

Note: Only the components with Eigenvalue > 1 are showed
